# The Application of Poly(2-(hydroxymethyl)acrylic Acid as a Functional Nanomaterial to Ensure the Biosafety of Herbal Decoctions

**DOI:** 10.3390/molecules30214276

**Published:** 2025-11-03

**Authors:** Yifei Guo, Xueqing Sun, Xiangsheng Zhao, Xiangtao Wang, Meihua Han, Zhengqi Dong

**Affiliations:** 1 Hainan Branch of the Institute of Medicinal Plant Development, Chinese Academy of Medical Sciences & Peking Union Medical College, Haikou 570311, China; 2 State Key Laboratory for Quality Ensurance and Sustainable Use of Dao-di Herbs, Institute of Medicinal Plant Development, Chinese Academy of Medical Sciences & Peking Union Medical College, No. 151, Malianwa North Road, Haidian District, Beijing 100193, China; sunxueqing@leadingpharm.com (X.S.);

**Keywords:** nanoadsorbents, Cd^2+^ ions, removal rate, biosafety, herbal decoctions

## Abstract

Heavy metal ions in herbal medicine sometimes exceed the standard limit, inducing severe and harmful problems in human health. Exploring new nanomaterials to chelate heavy metal ions and reduce their concentration in herbal decoctions could be a solution route. In this study, the nanoadsorbent poly(2-(hydroxymethyl)acrylic acid (PHMAA) was prepared via free radical polymerization and the hydrolysis method. PHMAA showed excellent dispersion in aqueous solution and self-assembled into spherical aggregates with a negative surface charge. After freeze-drying, PHMAA was a white solid powder with a loose porous structure. PHMAA presented no significant influence on the cell viability and weight of normal BALB/c mice. PHMAA showed good removal efficiency towards Cd^2+^ ions in aqueous solution; the removal rate exceeded 80%. In herbal decoctions, PHMAA presented moderate to good removal capacity towards Cd^2+^ ions; the removal rate was 60%, 83%, and 89% for the *Morindae officinalis radix* decoction, *Ligusticum wallichii* decoction, and *Coptidis rhizome* decoction, separately. When the concentration of Cd^2+^ ions in the decoction was decreased to 5 μg/mL, PHMAA also presented good removal efficiency. During the removal process, PHMAA played no influence on the active ingredients. To conclude, PHMAA showed good biosafety and removal capacity towards Cd^2+^ ions, which might be utilized as nanoadsorbents to reduce the concentration of Cd^2+^ ions in aqueous solution and herbal decoctions.

## 1. Introduction

Metal elements are related to several physiological activities and maintain the normal physiological functions in human health [1], while excessive metal ions could result in serious health problem [2,3]. It is reported that plants uptake heavy metal ions in the ionic or chelating formation, which would be transferred into the human body and induce severe side effects [4,5]. Chinese herbal medicine present good therapeutic effects in clinic and is utilized broadly. As a kind of plant, herbs also uptake and accumulate heavy metal ions [6,7]. The heavy metal content in several herbal medicines is reported to exceed the maximum permissible limits [8,9], which could induce a potential health problem.

To reduce their concentration, several technologies have been developed to remove the heavy metal ions in wastewater [10,11], including chemical precipitation [12], photocatalysis [13], electrochemical treatment [14], membrane separation [15], ion exchange [16], coagulation and flocculation [17], reverse osmosis [18], and so on. These methods are convenient and have been applied successfully in treating wastewater. Among these removal technologies, the adsorption method is considered more advantageous for high removal efficiency and low economic consumption [19,20]. As an analogue of adsorbents to remove heavy metal ions, organic polymeric materials have been explored and applied broadly [21] due to their abundant surface area, modified functional group, pore size distribution, and biocompatibility [22]. These organic polymeric materials contain abundant active groups, including carboxyl groups, amine groups, hydroxyl groups, and sulfhydryl groups, which could be chelated with metal ions to form stable complexes. Among these organic polymers, chitosan [23,24], cellulose [25,26], polysaccharide [27,28], starch [29,30], and their derivatives are utilized broadly due to the moderate to good removal efficacy towards different heavy metal ions in wastewater treatment. However, most of the reported polymeric materials show several disadvantages, including complicated methods, low removal efficiency, and complex work-up procedures.

Heavy metal ions not only exist in the wastewater but are also detected in herbs due to inevitable environmental pollution. To purify herbal decoctions, some nanoadsorbents are applied, different from the water solution, as herbal decoctions are a complex system, containing a lot of active ingredients. Several nanoadsorbents that are utilized in wastewater successfully are inappropriate to apply in decoction due to the unsatisfactory removal rate and limited types of metal ions; additionally, the active ingredients in herbal decoctions might be adsorbed by these removal materials. Therefore, the relative research is insufficient. A starch-based adsorbent was synthesized and applied to remove the Pb^2+^ and Hg^2+^ ions from four herbal decoctions, with the removal rate ranging from 50% to 89% [31]. The derivative of a cellulose binding module was prepared to remove lead from the decoction of *Honeysuckle*, and the results were that this nanoadsorbent had high efficiency for Pb^2+^ removal [32]. Also, several organic–inorganic hybrid materials were explored and utilized to adsorb heavy metal ions in decoctions. Polyethylene glycol-functionalized hybrid material could increase the efficient removal of heavy metal ions in *Ligusticum chuanxiong Hort*; the removal efficiency for Cd^2+^ and Pb^2+^ ions was 88% and 84%, respectively [33].

Effective nanoadsorbents that are suitable to be applied in decoctions should have two properties: high removal rate and biosafety. To enhance the removal efficacy, the nanoadsorbents should present high specific surface area and abundant active chelating sites. According to these properties, a series of nanoadsorbents based on poly(methacrylate citric acid) was designed to remove the Cu^2+^ and Pb^2+^ ions in different decoctions in our previous study [34,35]. These nanoadsorbents presented excellent solubility in aqueous solution, which guaranteed that the active site could be chelated with heavy metal ions effectively. After adsorbing heavy metal ions, precipitates were formed and could be separated via filtration from the system. Based on these results, it could be seen that nanoadsorbents with excellent water solubility and active sites presented a potential application to reduce the concentration of heavy metal ions in herbal decoctions.

To expand the types of nanoadsorbents and enhance the removal rate, poly(2-(hydroxymethyl)acrylic acid (PHMAA) was designed and utilized as a nanoadsorbent to remove heavy metal ions in herbal decoctions in this study. Free radical polymerization and the hydrolysis method were used to prepare the nanoadsorbent, which was applied in water and herbal decoctions to determine the removal efficiency. Combining the carboxyl group and hydroxyl group, PHMAA with good biosafety could remove Cd^2+^ ions with moderate to high adsorptive efficacy in water and three herbal decoctions, and the relative active ingredients were not affected.

## 2. Results and Discussion

### 2.1. Synthesis of Nanoadsorbent PHMAE

**Figure 1:**
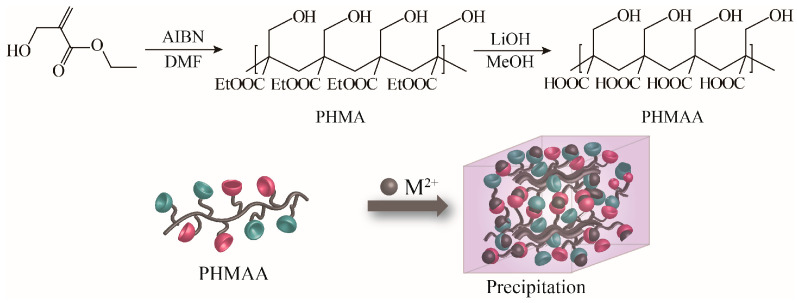
Synthesis scheme of PHMAA.

**Figure 2:**
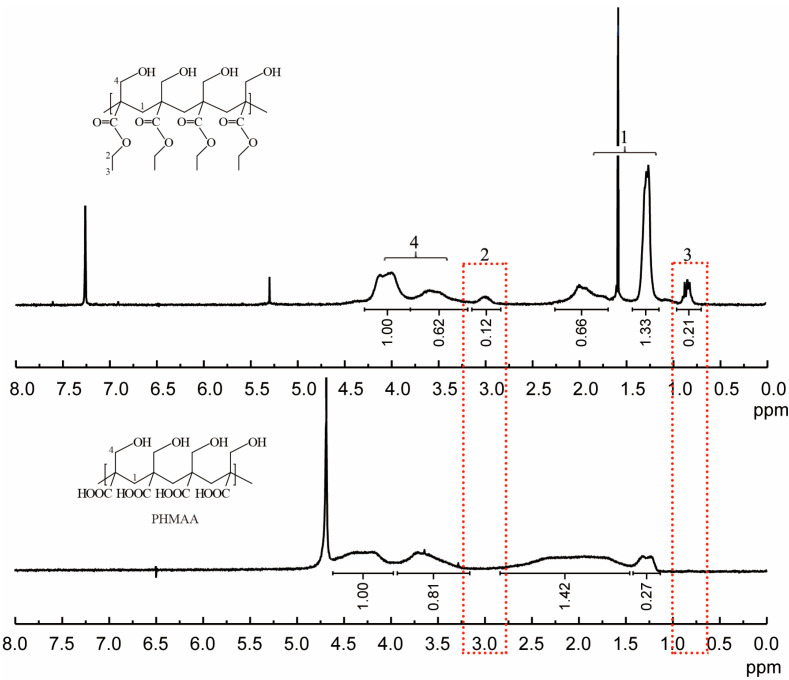
^1^H NMR spectra of PHMA (**up**) and PHMAA (**down**).

2-(hydroxymethyl)acrylic acid ethyl ester was utilized as the original monomer to prepare the polymer (PHMAE) via radical polymerization technology (Figure 1). The polymer was obtained as white powder with a yield of 76%. After hydrolysis with LiOH, the resultant nanoadsorbent poly(2-(hydroxymethyl)acrylic acid (PHMAA) was yielded as a white powder with a yield of 56%. After polymerization and hydrolysis, the chemical structures of PHMA and PHMAA were confirmed via ^1^H NMR spectra, which are shown in Figure 2. For PHMA, the signals of –CH_2_ (marked as 2) and –CH_3_ (marked as 3) appeared at 3.0 and 0.8 ppm, separately. These two peaks disappeared completely after hydrolysis, indicating that PHMAA was obtained as expected. Additionally, PHMAA presented excellent aqueous solubility, and the molar mass (Mn) was 27,297.

### 2.2. Particle Size and Morphology of PHMAA

**Figure 3:**
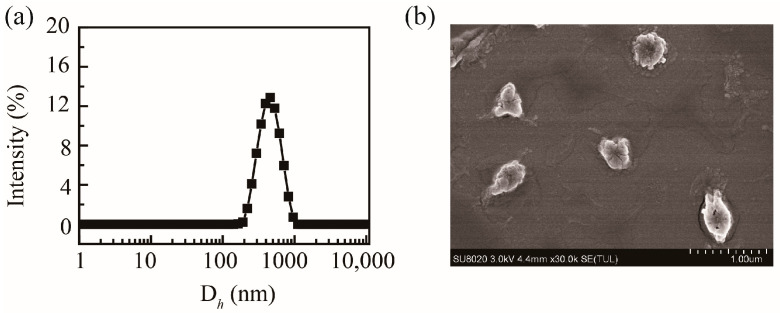
Particle size distribution curve of PHMAA (1 mg/mL) in aqueous solution (**a**) and SEM image (**b**).

Based on the abundant carboxyl groups and hydroxyl groups, PHMAA showed excellent aqueous solubility. PHMAA powder was dissolved in deionized water directly to form a homogeneous and transparent solution, and the concentration was 1 mg/mL. The mean hydrodynamic diameter of PHMAA in aqueous solution was approximately 760.3 ± 20.6 nm (PDI = 0.52 ± 0.06), and the particle size distribution curve is shown in Figure 3a. Due to the carboxyl groups and hydroxyl groups, the zeta potential was −32.7 ± 5.1 mV, allowing for chelation with positive metal ions. The morphology of PHMAA was detected by SEM, and the image is shown in Figure 3b. From the SEM image, it was revealed that PHMAA aggregates into spherical-like particles in aqueous solution.

### 2.3. Biosafety Test

**Figure 4:**
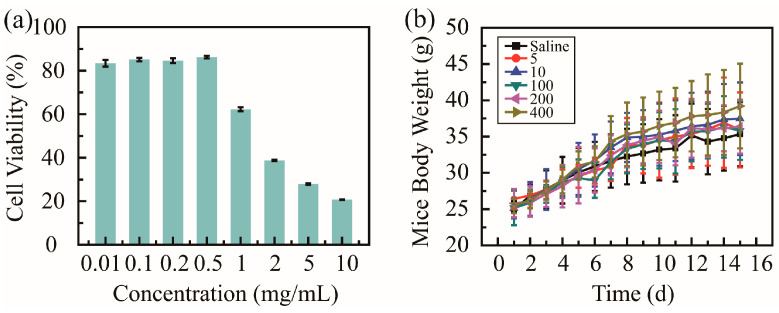
Biosafety of nanoadsorbent PHMAA: cell viability against HUVECs (**a**) and mouse body weight (**b**).

Cell cytotoxicity tests in vitro and animal experiments in vivo were utilized to evaluate the biosafety of PHMAA. Cytotoxicity against HUVECs was studied via an MTT assay, and the cell viability was recorded and is shown in Figure 4a. The cell viability presented two different tendencies; when the concentration was below 0.5 mg/mL, the cell viability was over 90%. These results suggest that PHMAA shows good biosafety in vitro at a low concentration. When PHMAA was utilized to remove heavy metal ions, the concentration of PHMAA was 0.2 mg/mL. Furthermore, most of the PHMAA was precipitated together with heavy metal ions, and trace PHMAA in solution is present. Considering the extremely low concentration in solution, PHMAA presents good biosafety in vitro.

When the body weight of BALB/c mice exceeded 20 g, these mice were divided into six groups (*n* = 10). All mice were treated with saline and PHMAA solution (concentration ranging from 5 to 400 mg/kg of mice body weight) via gastrointestinal administration every two days; meanwhile, the body weight were measured (Figure 4b). The body weight showed an increasing tendency, and no significant difference was shown between these six groups. Additionally, during the whole treatment procedure, all mice exhibited normal physiological conditions, as no signs of distress (unresponsive, labored breathing, and discharge) were observed. All these results indicates that PHMAA presents good biosafety in vivo. Although PHMAA is a non-natural polymer, it presented similar biosafety as previous results [36,37], which could be applied as nanoadsorbents.

### 2.4. Removal of Heavy Metal Ions in Aqueous Solution

**Figure 5:**
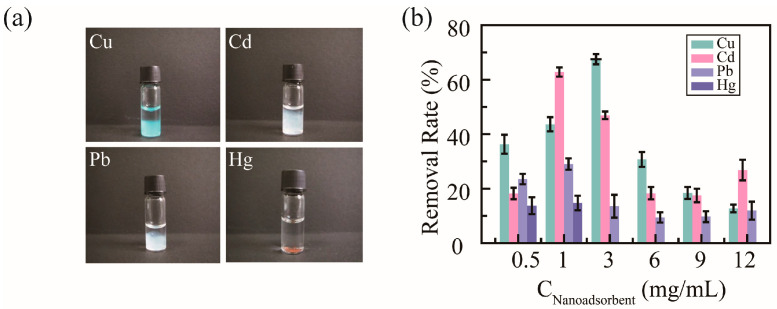
Images of PHMAA in different metal ion solutions (**a**) and removal rate in a series of PHMAA solutions (**b**).

To detect the removal efficacy of nanoadsorbents for heavy metal ions, PHMAA was utilized to chelate with Cu^2+^, Cd^2+^, Pb^2+^, and Hg^2+^ in neat aqueous solution. After adding PHMAA solution into the metal ion solution, precipitation presented immediately, and the images are shown as Figure 5a. For these four metal ions, precipitation resulted in different colors. The light blue precipitate occurred in Cu^2+^ ion solution, white precipitates are shown for Cd^2+^ and Pb^2+^ ion solutions, and a brown precipitate is shown for Hg^2+^ ion solution; all these color are consistent with the character of the heavy metal ions. Based on the level of precipitation, it can be seen that PHMAA shows better removal ability towards Cu^2+^ and Cd^2+^ ions.

Then, the removal ability of PHMAA was studied systematically, and the results are shown in Figure 5b. The initial concentration of metal ions was 100 μg/mL, and PHMAA at different concentrations, including 0.5, 1, 3, 6, 9, and 12 mg/mL, was added into the metal ion solution. After filtrating the precipitates, the residual concentration of heavy metal ions was detected by ICP mass spectrometry, and the removal rate was calculated. For all metal ions, PHMAA presented similar removal tendencies with an increasing concentration, which was increased first and then decreased. PHMAA showed a higher removal rate towards Cu^2+^ ions and Cd^2+^ ions. For Cu^2+^ ions, when the concentration of PHMAA was 3 mg/mL, the removal rate was approximately 80%. For Cd^2+^ ions, PHMAA presented the best removal ability at a concentration of 1 mg/mL, and the removal rate was approximately 75%. By increasing the concentration of PHMAA further, the removal rate decreased sharply. This phenomenon could be explained by the interaction between PHMAA molecules and ions. By increasing the concentration of PHMAA, the density of carboxyl group was increased, which led to unexpected disadvantages. Due to the hydrophilicity of free carboxyl groups, the hydrophilicity of the complex formed by PHMAA and metal ions is enhanced. Additionally, the repulsion interaction of free carboxyl groups between PHMAA chains make it difficult to form supramolecular aggregates via metal ions acting as the linker. These two factors would make it difficult to form stable precipitates and result in a low removal rate.

**Figure 6:**
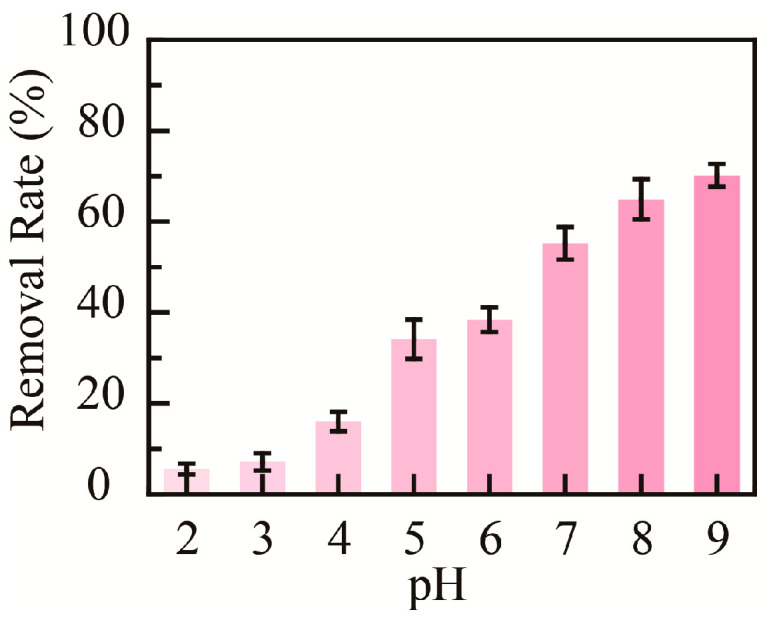
Removal rate of PHMAA at different pH conditions.

The medium’s pH value could affect the adsorptive capacity of nanomaterials; hence, the influence of pH on the adsorption properties of PHMAA for Cd^2+^ ions was studied. Briefly, PHMAA aqueous solution (3 mg mL^−1^) and Cd^2+^ ion aqueous solution (0.10 mg mL^−1^) with a pH value ranging from 2 to 9 were prepared. After 0.1 mL of PHMAA solution was added to 1 mL of Cd^2+^ ion solution and stirred, precipitation occurred at a higher pH value. The Cd^2+^ ion concentration in filtrate was detected by ICP mass spectrometry, and the removal rate at different pH values was calculated (Figure 6). By increasing the medium’s pH value from 2 to 9, the removal rate increased from 5% to 70%. This phenomenon could be explained by the ionization degree of the chelation site. The carboxyl group of PHMAA is occupied at the H^+^ state and is non-ionized at a lower pH. By increasing the pH, the carboxyl group presents an ionized state, which shows the desired chelation capacity [38,39,40]. The relative description and reference have been added into the renewed manuscript.

**Figure 7:**
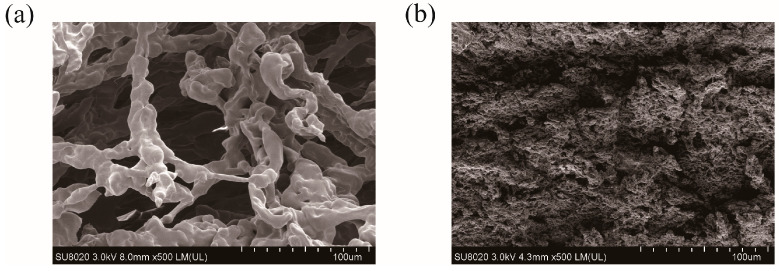
SEM images of PHMAA (solid powder (**a**) and precipitate after chelating with Cd^2+^ ions (**b**).

SEM images are taken to record the morphological changes between PHMAA and the precipitates. PHMAA showed a loose fiber-like morphology with a lot of pores (Figure 7a) [41], and, after chelating with metal ions, the structure changed significantly. The precipitates presented a tight structure (Figure 7b), and the loose pores disappeared. The morphological change revealed that chelation occurred between PHMAA and metal ions. During this procedure, metal ions performed as the “bridge” to connect the PHMAA chains, resulting in a sharply increased molar weight and decreased solubility; hence, the precipitates were formed.

### 2.5. Removal of Cd^2+^ Ions in Herbal Decoctions

Several nanomaterials are applied as nanoadsorbents to remove the heavy metal ions in aqueous solution [42,43,44]; however, the research to remove heavy metal ions in decoctions is rare. To ensure biosafety and human health, nanoadsorbents should be explored in the removal of trace heavy metal ions in herbal decoctions.

According to the results of the removal efficiency of PHMAA in aqueous solution, the removal capacity of PHMAA towards Cd^2+^ ions in herbal decoctions was detected systematically. The decoctions of *Ligusticum wallichii*, *Coptidis rhizome*, and *Morindae officinalis radix* containing Cd^2+^ ions were utilized as the model samples, and PHMAA solutions with different concentrations were added into these decoctions. After filtrating the precipitates, the concentration of Cd^2+^ ions was measured, and the removal rate was calculated (Table 1). PHMAA showed a moderate to good removal rate of Cd^2+^ ions in these three decoctions. Also, the removal rate was positively correlated with the concentration of PHMAA, which increased with an enhanced concentration of PHMAA. For *Coptidis rhizome*, PHMAA presented the highest removal capacity: almost 83% Cd^2+^ ions were removed. For *Ligusticum wallichii*, PHMAA presented a similar removal capacity: approximately 89% Cd^2+^ ions were adsorbed when 6 mg/mL PHMAA was added, furthering increasing the concentration, and the removal rate was decreased, which was consistence with the results in aqueous solution. PHMAA presented a moderate removal rate in *Morindae officinalis radix* decoctions: the removal rate was approximately 60%. Based on these results, it could be seen that PHMAA could effectively remove the Cd^2+^ ions in herbal decoction with a moderate to good removal efficiency.

**Figure 8:**
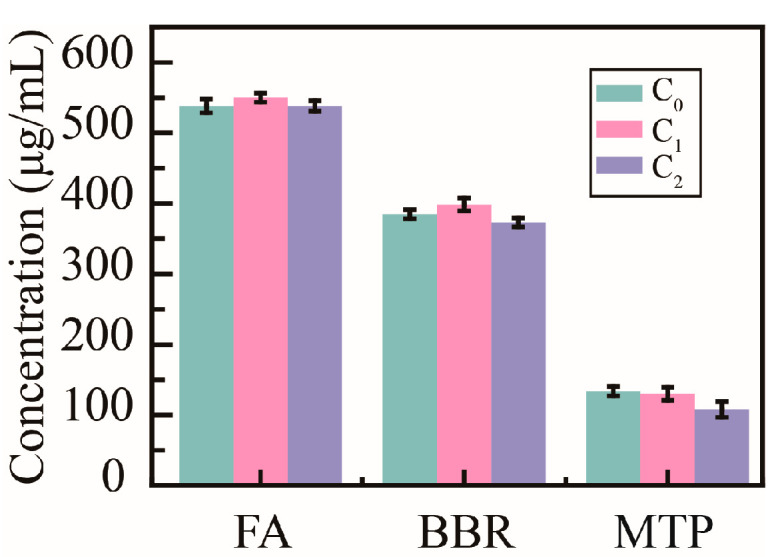
Concentration of active compounds in decoctions, *n* = 3.

To verify the influence of PHMAA on the active ingredients in decoctions, the concentrations of ferulic acid (FA), berberine (BBR), and monotropein (MTP) were determined (Figure 8). The original concentration (C_0_) of FA in the *Ligusticum wallichii* decoction was 538.0 ± 9.6 μg/mL; the concentration (C_1_) was 545.1 ± 6.5 μg/mL after adding Cd^2+^ ions; the final concentration (C_2_) was 538.3 ± 7.5 μg/mL after adding PHMAA and filtrating the precipitates. The initial concentration (C_0_) of BBR in the *Coptidis rhizome* decoction was 384.7 ± 6.5 μg/mL; the concentration (C_1_) was 398.5 ± 9.0 μg/mL after adding Cd^2+^ ions; the final concentration (C_2_) was 373.1 ± 6.6 μg/mL after adding PHMAA and filtrating the precipitates. Moreover, the initial concentration (C_0_) of MTP in the *Morindae officinalis radix* decoction was 133.5 ± 6.6 μg/mL; the concentration (C_1_) was 129.9 ± 9.5 μg/mL after adding Cd^2+^ ions; the final concentration (C_2_) was 107.7 ± 11.2 μg/mL after adding PHMAA and filtrating the precipitates. No significant change was shown during the whole removal procedure. This research implied that PHMAA would not affect the activity of decoctions.

### 2.6. Removal of Cd^2+^ Ions at Low Concentration in Herbal Decoctions

Furthermore, the removal efficiency of PHMAA towards Cd^2+^ ions at a low concentration in decoctions was studied. Considering the removal efficiency, the concentration of PHMAA was set as 6 mg/mL; the concentration of Cd^2+^ ions was selected as 5, 10, and 20 μg/mL, and the results are shown in Table 2. PHMAA presented good removal capacity in the *Ligusticum wallichii* decoction and *Coptidis rhizome* decoction: the removal rate was approximately 69% and 74%, separately. After adding PHMAA solution, Cd^2+^ ions were removed effectively, especially for the low concentration. When the original concentration of Cd^2+^ ions was 5 μg/mL, the final concentration was 1.73 and 1.21 μg/mL in the *Ligusticum wallichii* decoction and *Coptidis rhizome* decoction, separately. Although Cd^2+^ ions could not be removed completely, the residual concentration almost achieved the safety standard [45]. These results verified that PHMAA showed good removal efficiency towards Cd^2+^ ions in herbal decoctions.

Based on these results, it could be seen that PHMAA showed good removal capacity towards Cd^2+^ ions in decoctions and would not affect the activity of decoctions.

## 3. Materials and Methods

### 3.1. Materials

Methyl 2-(hydroxymethyl) acrylate was obtained from Shanghai Macklin Biochemical Technology Co., Ltd. (Shanghai, China). Heavy metal ion solution was purchased from the National Institute of Metrology, China. *Ligusticum*, *Coptis*, and *Morindae officinalis* were purchased from Beijing Tong Ren Tang Co., Ltd. (Beijing, China). Azobisisobutyronitrile (AIBN), triethylamine (TEA), and N,N-dimethylformamide (DMF) were purchased from Merck (Rahway, NJ, USA) and treated according to previous paper [34,35]. Other reagents were purchased and used directly without further purification.

### 3.2. Cells and Animals

The HUVEC cell line was purchased from the cell center of Peking Union Medical College and cultured according to published papers [34]. BALB/C mice (20 ± 2 g, 6–8 weeks) were purchased from Vital River Laboratory Animal Technology Co., Ltd. (Beijing, China). According to the Guidelines and Policies for Ethical and Regulatory for Animal Experiments approved by the Institute of Medicinal Plant Development (Approval Code: SYXK2017-0020; Approval Date:16 June 2017–16 June 2022), all mice were raised with food and water at room temperature for one week prior to experimentation (Approval No.: SLXD-2019111106).

### 3.3. Synthesis of PHMAE

A Schlenk tube was utilized to conduct free radical polymerization, in which ethyl 2-(hydroxymethyl)acrylate (0.50 g, 3.84 mmol) and AIBN (2.50 mg) were dissolved into 0.5 mL of anhydrous DMF. The mixture was thorough deoxygenated and then stirred at 60 °C. Methanol was added to terminate the reaction. After purifying by column chromatography (100–200 mesh silica gel, with dichloromethane as eluent), a transparent solid, PHMAE, was obtained (0.38 g, 76%).

### 3.4. Hydrolysis of PHMAA

PHMAE (0.50 mg) and LiOH (0.24 g) were added into the mixed solvent of H_2_O (15 mL) and methanol (50 mL) at 0 °C. The crude product was stirred overnight and purified via dialysis (MWCO = 8000–14,000) against deionized water to yield PHMAA as white powder (0.28 g, 56%).

### 3.5. Nuclear Magnetic Resonance (NMR) Characterization

PHMA was dissolved in CDCl_3_, and PHMAA was dissolved in D_2_O. These sample solutions were detected using a Bruker 600 MHz spectrometer to record NMR spectra. Detection was carried out at room temperature, and the scanning circulation was 16 times.

### 3.6. Molecular Weight

Gel permeation chromatography (GPC) was utilize to determine the molecular weight of the polymers. Detection was carried out on a Schimadzu GPC LC20 with an RID-20 refractive index detector at 35 °C. A single column was utilized; water (0.1 N NaNO_3_ and 0.06% NaN_3_) was used as the eluent. Polyethylene oxide (PEO) was applied as the standard polymer to obtain a calibration curve.

### 3.7. Dynamic Light Scattering Measurements

Dynamic light scattering (DLS) analysis (Zetasizer Nano-ZS analyzer, Malvern Instruments, Malvern, UK) was utilized to detect the particle size, polydispersity index, and surface potential. Each sample solution (1 mg mL^−1^) was measured with backscattering detection (θ = 173°) at room temperature; the measurement was performed 3 times.

### 3.8. Scanning Electron Microscopy

The sample solution and freeze-dried powder were placed on the copper column. Then, these samples were sputter-coated by gold–palladium (Au/Pd) for 1 min to obtain the conductive samples. Scanning electron microscopy (S-4800, Hitachi, Tokyo, Japan) was used to observe the morphology of the conductive samples, and the accelerating voltage was set as 30 mV; the images were recorded.

### 3.9. In Vitro and In Vivo Test

An MTT assay was performed to evaluate the biosafety of PHMAA. HUVECs were seeded in 96-well plates (10^4^/well) and cultured according to a previous paper [34]. Normal saline and a series of PHMAA solutions (0.01, 0.1, 0.2, 0.5, 1, 2, 5, and 10 mg mL^−1^) were added and incubated for another 48 h. After adding MTT solution (5 mg mL^−1^ in PBS, 20 μL) and incubating for 4 h, DMSO (150 μL) was added. An ELISA plate reader was used to record the absorbance of the samples; the wavelength was 570 nm. The cell viability was calculated according to the formula as follows:

Cell viability rate = OD_test_/OD_control_ × 100%. All assays were carried out in quintuplicate.

The normal BALB/c mice were randomly divided into 6 groups (10 mice per group). Mice were treated with normal saline (control group) and PHMAA solution (5 mg kg^−1^ to 400 mg kg^−1^ per mouse, test groups) by gastrointestinal administration for two days. The body weights of the mice were monitored every two days as an index of systemic toxicity.

### 3.10. Adsorption Measurement

PHMAA solution with a series of concentrations at 0.5–12 mg/mL were added into different metal ion solutions (100 μg/mL). The mixed solutions were stirred for 30 min, and turbidity or precipitation was shown during the stirred process. The precipitate was collected and freeze-dried after filtration. According to previous papers [34,35], the concentration of metal ions in filtrates was measured via inductively coupled plasma (ICP) spectrometry (Agilent ICP OES 730).

### 3.11. Adsorption Measurement in Chinese Herbal Decoctions

Herbal decoctions were prepared according to the *Chinese Pharmacopoeia* [45]. After obtaining these decoctions, metal ions were added to yield the original decoction samples. Then, the PHMAA solutions (0.5–9.0 mg/mL) were added, and the precipitates were filtrated after 0.5 h. The metal ion concentration and relative concentration of active ingredients in the filtrate were detected by ICP mass spectrometry and HPLC.

### 3.12. Statistical Analysis

To evaluate the differences between groups, SPSS 19.0 software (USA) was utilized, and the analysis method was one-way analysis of variance (ANOVA), during which *p* < 0.05 corresponded to statistical significance.

## 4. Conclusions

Though free radical polymerization and the hydrolysis method, the nanoadsorbent poly(2-(hydroxymethyl)acrylic acid (PHMAA) was synthesized successfully with a moderate total yield. PHMAA dispersed well in deionized water and formed aggregates with an irregular morphology; the hydrodynamic diameter and the zeta potential was 760 nm and −32.7 mV, correspondingly. PHMAA showed good biosafety, and no significant toxicity was shown in the cell inhibition test and animal experiment. In aqueous solution, PHMAA showed concentration-dependent removal efficiency towards Cu^2+^ and Cd^2+^ ions, which ranged from 75% to 80%. After chelating with metal ions, the loose fiber-like morphology of PHMAA changed to a tight solid. In herbal decoctions, PHMAA presented good removal capacity towards Cd^2+^ ions, especially at a low concentration, and the removal rate of PHMAA ranged from 60% to 88%; no influence on active ingredients in these decoctions were observed. These results revealed that PHMAA, having good biosafety, could remove Cd^2+^ ions in aqueous solution and herbal decoctions, which could potentially be applied as a nanoadsorbent to remove Cd^2+^ ions in wastewater or herbal medicine preparations.

## Figures and Tables

**Table 1:** Removal rate of Cd^2+^ ions in herbal decoctions ^a^ (*n* = 3).

Entry	C_PHMAA_ (mg/mL)	*Ligusticum wallichii* (%)	*Coptidis* *rhizome* (%)	*Morindae* *officinalis* (%)
1	0.5	56.6 ± 3.9	72.4 ± 2.5	21.9 ± 3.5
2	1	61.3 ± 5.4	84.9 ± 6.1	25.6 ± 2.1
3	3	74.0 ± 4.7	87.9 ± 4.4	61.9 ± 5.8
4	6	83.9 ± 5.0	88.6 ± 3.6	61.5 ± 3.9
5	9	61.2 ± 4.6	88.9 ± 2.9	62.0 ± 5.6

^a^ The original concentration of Cd^2+^ was 100 μg/mL.

**Table 2:** Removal rate of Cd^2+^ ions with low concentration in Chinese herbal decoction.

Entry	C_Cd_ (μg/mL)	*Ligusticum wallichii*			*Coptidis* *rhizome*		
		C_0_^a^ (μg/mL)	C_1_^b^ (μg/mL)	RR ^c^ (%)	C_0_^a^ (μg/mL)	C_1_^b^ (μg/mL)	RR ^c^ (%)
1	5	5.63	1.73	69.3	5.12	1.21	76.4
2	10	11.45	3.53	69.2	10.89	2.75	74.7
3	20	24.04	7.39	69.2	21.41	5.98	72.1

^a^ C_0_: original concentration in decoctions; ^b^ C_1_: final concentration in decoctions; ^c^ removal rate.

## Data Availability

The original contributions presented in this study are included in the article. Further inquiries can be directed to the corresponding author(s).
